# Effects of the Oxygen-Carrying Solution OxyVita C on the Cerebral Microcirculation and Systemic Blood Pressures in Healthy Rats 

**DOI:** 10.3390/jfb5040246

**Published:** 2014-11-18

**Authors:** Rania Abutarboush, Chioma Aligbe, Georgina Pappas, Biswajit Saha, Francoise Arnaud, Ashraful Haque, Charles Auker, Richard McCarron, Anke Scultetus, Paula Moon-Massat

**Affiliations:** 1 NeuroTrauma Department, Naval Medical Research Center (NMRC), Silver Spring, MD 20910, USA; E-Mails: chioma.aligbe@med.navy.mil (C.A.); georgina.pappas@quinnipiac.edu (G.P.); biswajit.saha@med.navy.mil (B.S.); francoise.arnaud@med.navy.mil (F.A.); ashraful.haque@med.navy.mil (A.H.); charles.auker@med.navy.mil (C.A.); richard.mccarron@med.navy.mil (R.M.); anke.scultetus@med.navy.mil (A.S.); pfmoon@hotmail.com (P.M.-M.); 2 Department of Surgery, Uniformed Services University of the Health Sciences, Bethesda, MD 20895, USA

**Keywords:** cerebral microcirculation, hemoglobin-based oxygen carriers (HBOC), oxygen therapeutic, vasoconstriction, intravital microscopy

## Abstract

The use of hemoglobin-based oxygen carriers (HBOC) as oxygen delivering therapies during hypoxic states has been hindered by vasoconstrictive side effects caused by depletion of nitric oxide (NO). OxyVita C is a promising oxygen-carrying solution that consists of a zero-linked hemoglobin polymer with a high molecular weight (~17 MDa). The large molecular weight is believed to prevent extravasation and limit NO scavenging and vasoconstriction. The aim of this study was to assess vasoactive effects of OxyVita C on systemic blood pressures and cerebral pial arteriole diameters. Anesthetized healthy rats received four intravenous (IV) infusions of an increasing dose of OxyVita C (2, 25, 50, 100 mg/kg) and hemodynamic parameters and pial arteriolar diameters were measured pre- and post-infusion. Normal saline was used as a volume-matched control. Systemic blood pressures increased (*P* ≤ 0.05) with increasing doses of OxyVita C, but not with saline. There was no vasoconstriction in small (<50 µm) and medium-sized (50–100 µm) pial arterioles in the OxyVita C group. In contrast, small and medium-sized pial arterioles vasoconstricted in the control group. Compared to saline, OxyVita C showed no cerebral vasoconstriction after any of the four doses evaluated in this rat model despite increases in blood pressure.

## 1. Introduction

The development of suitable cell-free hemoglobin-based oxygen carrying (HBOC) solutions has been an active area of research over the past three decades. These solutions are typically synthesized by various chemical modifications of hemoglobin derived from vertebrate or invertebrate blood or produced by bacteria using recombinant technology. HBOCs can be beneficial and potentially life-saving in the treatment of cerebral ischemia resulting from conditions of reduced cerebral blood flow. Several HBOC formulations have been shown to effectively reduce infarct volume in animal models of ischemic stroke [1,2,3], presumably due to enhanced oxygen transport to hypoxic tissue. Since ischemia is the secondary injury mechanism that contributes to neuronal damage after the initial traumatic insult in traumatic brain injury (TBI) [4], augmentation of oxygen delivery and exchange using an oxygen-carrying solution post-TBI could attenuate neuronal damage and improve outcome. Even though HBOCs have demonstrated the ability to fulfill hemoglobin’s major physiological role of reversibly binding oxygen and carbon dioxide, clinical side effects purportedly due to vasoconstriction plagued early development of these products [5].

The reaction of cell-free hemoglobin with vascular endothelial cell derived nitric oxide (NO) and the subsequent depletion of NO is the leading accepted hypothesis for the increases in systemic and pulmonary blood pressures observed after infusion of HBOC [6,7]. Elevation of mean arterial pressure (MAP) and renal glomerular filtration of hemoglobin in early animal studies using first generation HBOCs was likely caused by dissociation of hemoglobin tetramers into dimers that then extravasated through the endothelium lining and bound to vascular NO [7,8]. Another proposed factor that may contribute to the blood pressure side effects of HBOCs is the proximity of cell-free hemoglobin to endothelial cells (the sites of NO production) regardless of the degree of extravasation of the HBOC [9].

Over the years, chemical design modifications were employed in HBOC development to prevent vasoconstriction and renal filtration of cell-free hemoglobin. These modifications include polymerization and cross-linking with diaspirin (HemAssist, Baxter Health-care Corporation, Chicago, IL, USA) glutaraldehyde (Hemopure, HbO_2_ Therapeutics, Souderton, PA, USA; PolyHeme, Northfield Laboratories Inc, Evanston, IL, USA), or raffinose (Hemolink, Hemosol Inc., Toronto, Canada). Another modification has been conjugation of hemoglobin with a polyethylene glycol (PEG) shell (MP4CO, Sangart, San Diego, CA, USA; Sanguinate, Prolong Pharmaceuticals, South Plainfield, NJ, USA). These approaches aimed to increase the size of the HBOC molecule and prevent its dimerization and subsequent extravasation through the endothelium. This would, in turn, decrease binding of NO to hemoglobin in the HBOC, increase the bioavailability of NO and thereby prevent vasoconstriction. Some of these chemical modifications successfully reduced the extravasation of the HBOC and mitigated some of their vasoconstrictive effects, e.g., [10]; however, these chemically modified second-generation HBOCs still caused clinical side effects [11].

OxyVita C (OXYVITA Inc., New Windsor, NY, USA) is a newer generation HBOC produced by a patented “zero-linking” process of polymerization of bovine hemoglobin, which generates a large molecular weight tetrameric hemoglobin polymer molecule (average molecular weight ~17 MDa) and an average hydrodynamic radius of 360 Å. It has an oxygen affinity (P_50_) of 6 mm Hg [12] and retention time of about 3 hours in the rat [13]. The OxyVita hemoglobin can be stored at a wide range of temperatures (−80 to −45 °C) without changing its physiochemical properties and can be formulated in preparations using various physiological fluids (Lactated Ringer’s or saline [14]). This versatility makes this oxygen-carrier amenable for use in different situations including austere non-hospital military environments. Several investigations using polymeric zero-linked hemoglobin demonstrate that it has the functional properties of an effective oxygen therapeutic and is believed to lack vasoconstrictive and hypertensive properties [15,16]. Recently, Song *et al.* [17] showed that OxyVita C administered to normovolemic animals was not associated with vasoconstriction in skeletal muscle although a rise in systemic blood pressures was reported. Since vasoconstriction was one of the hurdles that halted regulatory approval of HBOCs, those preliminary results were promising. As our laboratory was interested in pursuing the use of an Oxygen Therapeutic (OT) for traumatic brain injury (TBI), the aim of this study was specifically to examine the effects of OxyVita C, using a step-wise dose design, on cerebral pial arterioles and systemic blood pressure in healthy rats.

## 2. Results and Discussion

### 2.1. General Observations

Animals in the normal saline (0.9% NaCl, *N* = 13, 404 ± 52 g) and OxyVita C (*N* = 14; 384 ± 30 g) groups survived the protocol without any adverse events. Body temperatures (monitored rectally) did not change over the course of the study and were 36.8 ± 0.6 and 36.6 ± 0.5 °C for the OxyVita C and 0.9% NaCl animals, respectively. Although heart rate (HR) was different between the groups pre-treatment (Table 1), HR did not change over time in the OxyVita C group and HR increased only after the third infusion in the 0.9% NaCl group (*P* = 0.015). This 0.9% NaCl increase was within accepted range of normal values (Table 1).

### 2.2. Systemic Blood Pressures 

**Figure 1:**
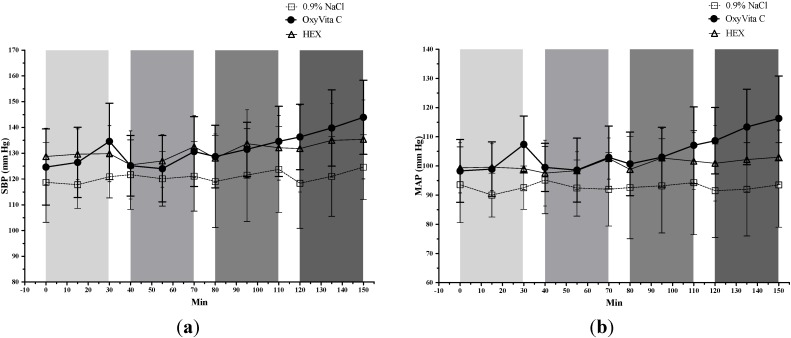
Systemic blood pressures (mean ± SD). (**a**) Systolic blood pressure (SBP) and (**b**) mean blood pressure (MAP). Animals were treated with OxyVita C (escalating doses), 0.9% NaCl, or HEX as four-30 min IV infusions represented as light to dark grey vertical bars.

Table 1 and Figure 1 provide individual systolic (SBP) and mean (MAP) as well as statistical comparisons of all infusions for the treatment groups. Diastolic blood pressure (DBP) data are presented in Table 1 only. These data include not only the OxyVita C and 0.9% NaCl groups but also data from a Hextend (HEX)-treated group. The HEX group was analyzed as a *post hoc* comparator to better interpret the blood pressure results from the OxyVita C-treated animals because this HEX group (used for another study) received twice the volume of HEX as OxyVita C. Overall, within the OxyVita C group, there was a significant (*P* ≤ 0.05) cumulative increase in systemic pressures (*i.e.*, comparing T0 to T150) as well as significant increases in either SBP, MAP, or both after each infusion. In contrast, there were no significant changes in blood pressures associated with infusion of 0.9% NaCl or HEX. Among the experimental groups, MAPs and/or SBP were significantly higher in the OxyVita C group than the 0.9% NaCl group after most/all of the four infusions. The OxyVita C group also had significantly higher MAPs than the HEX animals after most of the infusions. DBP data were not analyzed statistically, although changes in DBP were similar to the trends exhibited by MAP and SBP data.

**Table 1:** Blood pressure and heart rate parameters for OxyVita C, Hextend (HEX), and saline (0.9% NaCl) groups. All values are means ± SD. * Post-infusion value (T30, T70, T110 or T150) is significantly different (*P* ≤ 0.05) from its corresponding within-group value at T0; values at pre-infusion times T40, T80, and T120 were not compared to T0. ^#^ Post-infusion value (T30, T70, T110 or T150) is significantly different (*P* ≤ 0.05) from its corresponding within-group value at the preceding pre-infusion timepoint (T0, T40, T80, or T120). Significant differences (*P* ≤ 0.05) between treatment groups at each timepoint are indicated by similar superscripted letters (^a^ or ^b^).

Time	Treatment	SBP (mm Hg)	MAP (mm Hg)	DBP (mm Hg)	HR (beats/min)
T0 (Pre-treatment)	OxyVita C	124.6 ± 14.8	98.3 ± 10.7	82.3 ± 9.6	385.8 ± 76.3 ^a^
	0.9% NaCl	118.7 ± 15.5	93.5 ± 13.0	78.5 ± 11.8	304.5 ± 61.3 ^a,b^
	HEX	128.7 ± 11.0	99.4 ± 8.6	81.7 ± 9.9	391.6 ± 73.8 ^b^
T30 (After infusion 1)	OxyVita C	134.6 ± 14.8 ^#,a^	107.4 ± 9.7 ^#,a,b^	90.4 ±7.9	392.9 ± 72.8 ^a^
	0.9% NaCl	120.9 ± 8.2 ^a^	92.5 ± 7.4 ^a^	76.5 ± 7.6	308.3 ± 41.9 ^a,b^
	HEX	129.8 ± 10.9	99.1 ± 7.2 ^b^	80.7 ± 8.6	367.0 ± 79.2 ^b^
T40 (Before infusion 2)	OxyVita C	125.1 ± 11.7	99.5 ± 8.2	84.1 ± 8.2	388.5 ± 90.0
	0.9% NaCl	121.7 ± 13.5	95.2 ± 11.5	79.0 ± 11.1	338.0 ± 35.8
	HEX	125.5 ± 13.2	97.5 ± 11.3	79.7 ± 12.5	349.8 ± 89.4
T70 (After infusion 2)	OxyVita C	130.6 ± 13.6 ^#^	102.9 ± 10.8	84.8 ± 10.3	383.5 ± 78.9
	0.9% NaCl	121.1 ± 13.6	92.0 ± 12.6	76.7 ± 12.0	318.1 ± 43.7
	HEX	132.5 ± 12.0	102.5 ± 7.0	85.0 ± 4.8	363.6 ± 79.4
T80 (Before infusion 3)	OxyVita C	128.7 ± 12.1	100.7± 10.9 ^a^	83.9 ± 10.2	387.3 ± 97.1
	0.9% NaCl	119.0 ± 17.8	92.6 ± 17.5 ^a,b^	76.8 ± 16.5	335.8 ± 65.4
	HEX	128.1 ± 9.4	98.8 ± 6.2 ^b^	81.4 ± 5.9	377.7 ± 72.5
T110 (After infusion 3)	OxyVita C	134.6 ± 13.6 ^#^	107.1 ± 13.2 ^#,a^	90.1 ± 13.5	388.1 ± 98.9
	0.9% NaCl	123.7 ± 16.7	94.3 ± 17.8 ^a^	78.8 ± 17.3	331.0 ± 44.6 *
	HEX	132.1 ± 12.5	101.6 ± 9.8	83.5 ± 7.3	387.7 ± 76.6
T120 (Before infusion 4)	OxyVita C	136.3 ± 12.7	108.6 ± 11.4	91.6 ± 11.1	382.1 ± 87.2
	0.9% NaCl	118.3 ± 17.5	91.5 ± 16.0	75.3 ± 15.6	351.2 ± 47.9
	HEX	131.8 ± 16.9	100.9 ± 12.9	83.3 ± 10.8	370.7 ± 73.8
T150 (After infusion 4)	OxyVita C	143.9 ± 14.4 ^#,^*^,a^	116.3 ± 14.5 ^#,^*^,a,b^	98.9 ± 14.4	386.3 ± 84.8
	0.9% NaCl	124.6 ± 12.6 ^a^	93.5 ± 14.5 ^a^	77.1 ± 14.3	324.7 ± 56.5
	HEX	135.4 ± 15.3	103.0 ± 9.3 ^b^	84.8 ± 7.0	363.5 ± 68.3

Our data demonstrate that OxyVita C was associated with blood pressure increases but blood pressures were not affected by an equivalent volume of crystalloid (saline) or twice the volume of colloid (HEX). Even though OxyVita C was administered to normovolemic rats with a resultant ~10% increase in total blood volume with each infusion (assuming a total blood volume of ~20 mL), the increase in systemic blood pressures was not simply due to the infused volume because the volume-control saline group did not exhibit an equal increase in blood pressure. The fact that animals in the HEX group received twice the volume of a colloid with a higher oncotic pressure (~37 mm Hg) than that of OxyVita C (~3 mm Hg in lactated Ringer’s [16]) and that these HEX-treated animals still did not show an increase in blood pressures further suggests that the increase in systemic blood pressures in the OxyVita C group is not due to a simple increase in blood volume. Fluid redistribution, changes in cardiac function or vasoconstriction of large systemic vascular beds could be the underlying causes of the increase in blood pressure after OxyVita C but none of these could be evaluated in this screening study. Heart rate did not change following OxyVita C and cardiac output was not measured. The time between each of the four 30-min long infusions in this study was 10 min and the retention time of OxyVita C in the circulation is about 3 h; therefore, the effect of each of the OxyVita C doses is additive. Thus, the short time following each of the infusions was not sufficient for blood pressure in the OxyVita C animals to return to baseline values. In support of our findings, a previous intravital microscopy study using a similar design of sequential infusions of OxyVita C into normovolemic rats [17] also reported an increase in systemic blood pressures and without vasoconstriction in skeletal muscle arterioles.

### 2.3. Cerebral Pial Arteriolar Diameters

**Figure 2:**
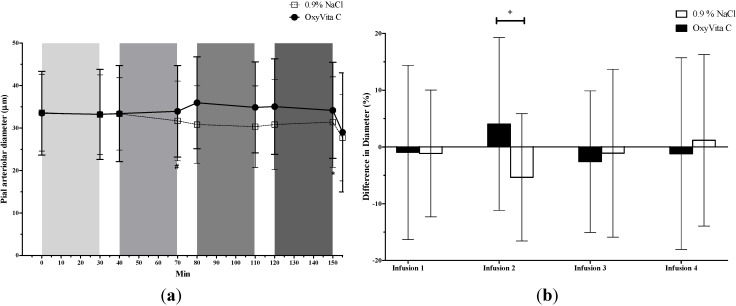
Diameter of small-sized (< 50 µm) pial arterioles (mean ± SD) from rats treated with OxyVita C (escalating doses) or 0.9% NaCl. (**a**) Vessel diameter. Test treatments were administered as four-30 min IV infusions represented as light to dark grey vertical bars. Final data point for each treatment is effect of topical BaCl_2_. * Indicates a significant (*P* ≤ 0.05) cumulative decrease in vessel diameters in the 0.9% NaCl group (comparing diameters at T0 to T150). ^#^ Indicates a significant (*P* ≤ 0.05) decrease in vessel diameters in the 0.9% NaCl group during the second infusion period (T70 *vs.* T40). (**b**) Percent change in vessel diameter during each infusion. ^+^ Indicates a significant difference (*P* ≤ 0.05) in percent change of vessel diameter between the two treatment groups (*P* ≤ 0.05).

For small-sized pial arterioles (<50 µm; Figure 2), there was a significant cumulative decrease in vessel diameters (comparing T0 to T150) in the 0.9% NaCl group, but there was no vasoconstriction in the OxyVita C group either cumulatively or during each of the four infusions. In addition, the small-sized arterioles significantly vasoconstricted in the 0.9% NaCl group during the second infusion but not during any of the other infusions. Between the two groups, the small-sized pial arterioles in the OxyVita C group were larger than the saline group only after the second infusion.

**Figure 3:**
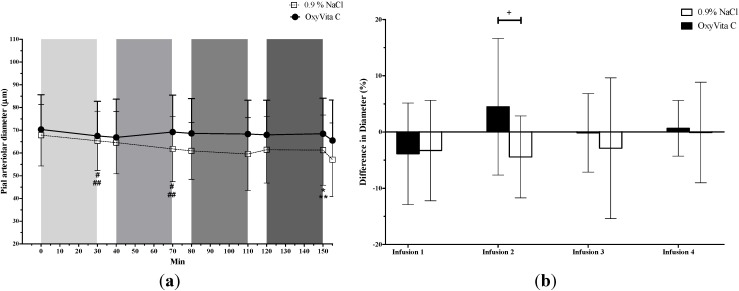
Diameter of medium-sized (50–100 µm) pial arterioles (mean ± SD) from rats treated with OxyVita C (escalating doses) or 0.9% NaCl. (**a**) Vessel diameter. Test treatments were administered as four-30 min IV infusions represented as light to dark grey vertical bars. Final data point for each treatment is effect of topical BaCl_2_. ** And * indicate a significant (*P* ≤ 0.05) cumulative decrease in vessel diameters in the OxyVita C and 0.9% NaCl (comparing diameters at T0 to T150), respectively. ^##^ And ^#^ indicate a significant (*P* ≤ 0.05) decrease in vessel diameters the OxyVita C and 0.9% NaCl, respectively, during the first and second infusion periods (T30 *vs.* T0 and T70 *vs.* T40). (**b**) Percent change in vessel diameter during each infusion. ^+^ Indicates a significant difference (*P* ≤ 0.05) in percent change of vessel diameter between the two treatment groups.

For medium-sized vessels (50–100 µm; Figure 3), there was a significant cumulative decrease in vessel diameters (comparing T0 to T150) in both OxyVita C and saline groups. In OxyVita C-treated rats, there was significant vasoconstriction during the first infusion but a significant vasodilation during the second infusion and no further changes during the third and fourth infusions. In saline-treated rats, there was significant vasoconstriction during both the first and second infusions and no further changes during the third and fourth infusions. Between the treatment groups, the only significant difference occurred after the second infusion and can be attributed to the concurrence of vasodilation in the OxyVita C group and vasoconstriction in the 0.9% NaCl group.

Infusion of 0.9% NaCl resulted in vasoconstriction in both small-sized and medium-sized microvessels. In contrast, small-sized vessels did not vasoconstrict after any dose of OxyVita C and the cumulative vasoconstriction observed in medium-sized vessels could be attributed to the vasoconstriction observed during the first dose (2 mg/kg) of OxyVita C. It is important to note that the two treatment groups were not different at this time point and that subsequent higher OxyVita C doses did not cause additional vasoconstriction. At the end of the study, all vessels constricted in response to topical application of aqueous barium chloride confirming the viability of the vessels and their responsiveness to any potential vasoconstrictive events. Thus, if OxyVita C were, itself, a vasoconstrictive compound, it is likely that further vasoconstriction would have been detected in this model. These observations indicate that the vasoconstriction following the lowest dose of OxyVita C was more likely an effect of the experimental preparation that was not off-set by OxyVita C vasodilation. This vasoconstriction is, in fact, similar to findings not only in the 0.9% NaCl group but also to results from another study conducted in this laboratory using the same cranial window model that showed a decrease in pial arteriolar vessel diameters in saline-treated rats [18]. These findings suggest the possibility that vasoconstriction may be an effect associated with the model and a result of the surgery, anesthesia, changes in intra-cranial pressure, or local changes in surface temperature of the brain. Interestingly, after the 25 mg/kg (second infusion) dose of OxyVita C, the medium-sized arterioles vasodilated. This vasodilation (as well as lack of vasoconstriction in the small-sized pial arterioles) may have been caused by the vasodilatory effects of carbon monoxide (CO) which is present in this OxyVita C formulation.

Our data clearly demonstrate that the rise in blood pressures after OxyVita C infusion is not associated with arteriolar vasoconstriction in the cerebral pial microcirculation of healthy rats. It is noteworthy that the response of cerebral vasculature to blood pressure changes may vary depending on whether the vessels are pial or intraparenchymal and the part of the brain (e.g., brainstem *vs.* cerebrum) in which the vessels are located [19]. Therefore, it is arguable that the response of intraparenchymal vessels to the modest 5–9 mm Hg blood pressure rise after OxyVita C might be different from that of the pial arterioles measured in the current study. It is unclear how a modest increase in blood pressure affects tissue oxygenation and future studies measuring tissue oxygenation should explore this question. In addition, recent computational models for predicting the relationships among pial vessel size, blood flow, tissue oxygenation and pial blood pressure [20] are important tools that can be used in future research for predicting tissue oxygen tension.

In contrast to our findings, Matheson and colleagues [15] reported vasoconstriction of pial arterioles without an increase in MAP after administration of a zero-link HBOC similar to OxyVita C (average molecular weight = 20MDa). Those authors proposed that the vasoconstriction of pial arterioles in their study was a regulatory response to prevent tissue over-oxygenation. However, in neither study was brain tissue oxygenation measured. Other reasons for the difference between the findings of that study and the present study are most likely due to model differences: the Matheson *et al.* [15] study used an isovolemic exchange transfusion hemodilution model while this study used a normovolemic model where infusions would increase vascular volume. Additionally, the presence of CO in our OxyVita C formulation (and not in their formulation) may also account for the observed lack of vasoconstriction as CO has been shown to vasodilate pial arterioles [21].

In a study similar to the present report, using a parallel design of sequential infusions of OxyVita C to normovolemic rats [17], an increase in systemic blood pressures without vasoconstriction in skeletal muscle arterioles was reported. Based on these two intravital microscopy studies, and with the understanding that the vasomotor response of pial microvessels to changes in systemic blood pressure may be different from that of skeletal muscle, the OxyVita C-induced increase in MAP and SBP does not appear to be caused by either pial cerebral or skeletal muscle arteriolar vasoconstriction. More invasive monitoring in larger species, to include systemic and pulmonary vascular resistances, is indicated to confirm this preliminary result in rats.

### 2.4. Laboratory Analyses

Overall, arterial blood data were within normal physiological ranges (Table 2). Total blood hemoglobin (Hb) was expectedly higher (*P* = 0.014) after OxyVita C due to the exogenous administration of hemoglobin. Similarly, the plasma hemoglobin was increased in the OxyVita C group (0.52 ± 0.08 g/dL) and was higher than the plasma hemoglobin of the 0.9% NaCl (0.03 ± 0.01 g/dL) group. All whole blood methemoglobin concentrations were too low to be detected (data not provided), but plasma methemoglobin was significantly higher for OxyVita C (12.39% ± 8.72%) compared to 0.9% NaCl at the end of the study. This plasma methemoglobin may be due to some of the OxyVita C product being oxidized from hemoglobin (functional) to methemoglobin (nonfunctional). Since brain tissue oxygenation was not measured in this study, it is unknown what effect this amount of plasma methemoglobin might have on the clinical effects of OxyVita C. There was an increase in lactate values after the third and fourth infusions of OxyVita C; however, this increase was neither statistically nor physiologically significant.

Urine samples collected at the end of the study were analyzed for hemoglobin content to determine if the product was filtered through the kidneys. Urine hemoglobin content of the OxyVita C-treated rats (0.004 ± 0.001 g/dL; *N* = 8) was equivalent to that of the 0.9% NaCl group (0.004 ± 0.001 g/dL; *N* = 3) and, hence, it can be concluded that the large polymeric hemoglobin molecule in the OxyVita C solution is not cleared from circulation by renal filtration.

**Table 2:** Arterial blood chemistry and gas parameters (mean ± SD). ^§^ Indicates that this post-infusion measurement (T30, T70, T110 or T150) is significantly (*P* ≤ 0.05) different from T0 within a treatment group. The other pre-infusion times (T40, T80, and T120) were not compared to T0. Treatment groups with the same superscripted letters (^a^ and ^b^) are statistically different from each other.

Time	Group	Hb (g/dL)	pH	PaCO_2_ (mm Hg)	PaO_2_ (mm Hg)	Lactate (mmol/L)
T0 (Pre-treatment)	OxyVita C	12.6 ± 1.0	7.389 ± 0.05	39 ± 8	132 ± 107	0.8 ± 0.7
	0.9% NaCl	12.5 ± 0.9	7.349 ± 0.08	47 ± 12	110 ± 41	0.6 ± 0.3
T30 (After infusion 1)	OxyVita C	12.3 ± 0.8	7.385 ± 0.07	37 ± 9	130 ± 110	0.8 ± 0.6
	0.9% NaCl	12.4 ± 0.9	7.352 ± 0.07	46 ± 10	116 ± 34	0.7 ± 0.3
T70 (After infusion 2)	OxyVita C	12.6 ± 0.6 ^a^	7.359 ± 0.05 ^§^	40 ± 7	146 ± 125	0.8 ± 0.5
	0.9% NaCl	12.3 ± 1.3 ^b^	7.354 ± 0.06	42 ± 9	118 ± 29	0.7 ± 0.3
T110 (After infusion 3)	OxyVita C	12.6 ± 1.1 ^a^	7.349 ± 0.05 ^§^	38 ± 6	133 ± 99	1.1 ± 0.4 ^a^
	0.9% NaCl	12.1 ± 1.1 ^b^	7.340 ± 0.06	43 ± 9	111 ± 31	0.7 ± 0.2 ^a^
T150 (After infusion 4)	OxyVita C	13.4 ± 1.2 ^§ a^	7.361 ± 0.06	39 ± 10 ^a,b^	155 ± 129	1.2 ± 0.4 ^a^
	0.9% NaCl	12.6 ± 1.1 ^b^	7.323 ± 0.05 ^§^	47 ± 8 ^a^	106 ± 29	0.7 ± 0.2 ^a^

## 3. Experimental Section 

The study protocol was reviewed and approved by the Walter Reed Army Institute of Research/Naval Medical Research Center Institutional Animal Care and Use Committee in compliance with all applicable Federal regulations governing the protection of animals in research.

### 3.1. Animal Preparation and Surgical Procedures

Healthy male Sprague-Dawley rats (*N* = 27) weighing 300–450 g (Charles River Laboratories, Wilmington, MA, USA) were anesthetized with a mixture of ketamine and acepromazine (72 and 4 mg/kg, respectively, intraperitoneal [IP]). Core body temperature was monitored rectally and maintained at 37 °C with a warming pad (Harvard Apparatus, Cambridge, MA, USA). The femoral vein was cannulated with PE-50 catheter for infusion of the HBOC and control solutions. A femoral artery was cannulated for blood sampling and arterial blood pressure monitoring (Datascope Corporation, Montvale, NJ, USA). Following vessel cannulation, all animals were intubated and mechanically ventilated with a small animal ventilator (RSP1002, Kent Scientific Corp., Lichtfield, CT, USA) to maintain normal blood gases (PaCO_2_ of 35–45 mm Hg and PaO_2_ ≥ 80 mm Hg). Access to pial microcirculation was gained through a rectangular craniotomy (~2 mm × 4 mm) prepared in the right parietal bone. The dura was cut and reflected to the side to allow visualization and imaging of pial surface microvasculature [22]. The surface of the brain was superfused with artificial cerebrospinal fluid (Na 150 mM, K 3.0 mM, Ca 1.4 mM, Mg 0.8 mM, P 1.0 mM, Cl 155 mM; Harvard Apparatus, MA, USA) to maintain electrolyte balance. A glass cover slip was used to cover the craniotomy to prevent drying of the brain surface. A stereomicroscope (SZ16, Olympus, Japan) equipped with a DP-73 digital camera was used for direct visualization and imaging of pial microvessels.

Pial arteriolar diameters were measured at the same locus throughout the experiment using the computer program CellSens (Olympus, 2010). All vessel images were acquired at 80× magnification. Prior to euthanasia, a 5% aqueous BaCl_2_ solution was applied topically as a positive control to validate vessel responsiveness as it has been shown to be a vasoconstrictor in cerebral blood vessels [23].

### 3.2. Test Solutions 

OxyVita C (OXYVITA Inc., New Windsor, NY, USA), is a large tetrameric bovine hemoglobin molecule. The concentration of hemoglobin is 6 g/dL and colloidal osmotic pressure (COP) is 3 mmHg. Given the low COP of the OxyVita C solution and the fact that it is dissolved in an isotonic balanced crystalloid, normal saline (0.9% NaCl; Abbott Laboratories, Chicago, IL, USA) was deemed a suitable volume control solution.

### 3.3. Experimental Protocol 

After instrumentation and stabilization, all data was collected for baseline (T0) determination. Rats were then randomized to the experimental (OxyVita C, *N* = 14) or control (0.9% NaCl, *N* = 13) groups. Each animal received four IV infusions of either the test or control solution, each infusion lasting 30 min, with 10 min between each infusion. The control or test solution volume administered per infusion was 2 mL/kg, which is equivalent to an infusion rate of 4 mL/kg·h^−1^. The OxyVita C solution was administered in an escalating dose sequence of 2, 25, 50 and 100 mg/kg of OxyVita polymer for doses 1–4, respectively,(for a cumulative dose of 177 mg/kg or 2.95 mL/kg.h^-1^ of OxyVita polymer) which is equivalent to 0.03, 0.42, 0.83, and 1.67 mL/kg·h^−1^ for those same respective infusions. For the OxyVita C animals, the infused volume of OxyVita solution was augmented with additional 0.9% NaCl administered via a separate IV catheter to bring the total fluid volume per infusion to 4 mL/kg·h^−1^, equivalent to the volume and rate of the 0.9% NaCl control animals. After study completion, and to better interpret blood pressure data from the OxyVita C animals, blood pressure data were also compared *post hoc* to a secondary group of animals (*N* = 11) that were administered the 6% hetastarch solution HEXTEND^®^ (HEX, Hospira, Inc., Lake Forest, IL, USA) at twice the volume (8 mL/kg·h^−1^ per infusion) of the OxyVita C and 0.9% NaCl groups.

Pial arteriolar vessel diameters, hemodynamic parameters (systolic, diastolic and mean arterial pressures), and vital signs (HR and temperature) were recorded at the beginning and end of each infusion. Arterial blood samples were obtained at baseline and at the end of each infusion. Blood samples were analyzed on an automated blood gas system (ABL 700, Radiometer, Copenhagen, Denmark) for pH, partial pressures of oxygen (PaO_2_) and carbon dioxide (PaCO_2_), lactate, and bicarbonate ($\text{HC}{\text{O}}_{3}^{-}$). Plasma hemoglobin and methemoglobin concentrations were determined at the end of the experiment using modified Drabkin’s [24] and modified Evelyn-Malloy [25,26] biochemical assays, respectively.

### 3.4. Statistical Analyses

Data analyses were performed using IBM SPSS Statistics 21.0 (IBM Corporation, Armonk, NY, USA, 2012). Mixed general linear models were used for analyzing heart rate, blood pressure, vessel diameter, temperature, blood gases, methemoglobin and lactate data, while rat body weights were analyzed using a one-way analysis of variance. Similar to previous cerebrovascular studies [18,27,28], the pial arteriolar vessel diameters were divided into three categories based on size at baseline measurement: (1) “small” vessels having a diameter <50 µm, (2) “medium” vessels with diameters of 50 to 100 µm, and (3) “large” vessels with diameters >100 µm. The choice of the size categories was based on the fact that the amount of smooth muscle increases with vessel size which could lead to differences in degree of vessel contraction [29]. Results are based on 11 to 14 rats per group, with the number of vessels ranging from 55 to 71 for small-sized, 42 to 56 for medium-sized, and 3 to 10 for large-sized vessels. For vessel diameters, percent change from baseline was compared among the three groups. Percent change was determined using pre- and post-infusion data for each infusion separately. Least square means were used for post hoc t-tests. Due to the small sample size for large-sized vessels (*N* = 3 for 0.9% NaCl and *N* = 10 for OxyVita C rats), these data were not analyzed further. For all parameters, a *P*-value ≤ 0.05 was considered statistically significant. 

## 4. Conclusions 

Infusion of OxyVita C did not cause vasoconstriction in cerebral pial arterioles in healthy rats although OxyVita C did cause an increase (~10 mmHg) in systemic blood pressures. Infusion of the control fluids resulted in no change in blood pressure and an overall vasoconstriction of cerebral pial arterioles. Future research into the usefulness of OxyVita C to deliver oxygen to brain tissue in traumatic brain injury models is warranted.
